# Antiunitary Symmetry in Non-Hermitian Dissipative Dynamics and Neutron Scattering

**DOI:** 10.3390/e28040404

**Published:** 2026-04-03

**Authors:** László Deák

**Affiliations:** 1 HUN-REN Wigner Research Centre for Physics, 1121 Budapest, Hungary; deak.laszlo@wigner.hun-ren.hu; 2 Department of Natural Science, Ludovika University of Public Service, 1083 Budapest, Hungary

**Keywords:** symmetry transformations, complex symmetric operator, scattering theory, arrow of time, reciprocity, polarized neutron reflectometry

## Abstract

Symmetry transformations are defined by operators in quantum mechanics that preserve the modulus of the scalar product between Hilbert space vectors. According to Wigner’s theorem, any such transformation is represented by either a unitary linear operator or an antiunitary (isometric conjugate-linear) operator. Although antiunitary symmetries—most notably time reversal and charge conjugation—are encountered less frequently than unitary ones, they are fundamental to the description of non-conservative and reversible systems. The most frequently treated antiunitary operators are the involutive ones, called conjugations. Any antiunitary operator can be written as a product of a conjugation and a unitary operator. Considering general scattering problems defined by a scattering potential and separating conjugation from the symmetry operator, one can find the role of complex symmetric (in other words self-transpose) unitary operators in physical problems. This approach provides a robust framework for analyzing the role of non-Hermitian symmetries in wave scattering and dissipative dynamics. To demonstrate the practical applicability of these theoretical concepts, we analyze the case of polarized neutron reflectometry (PNR). We show that the scattering potential in PNR, comprising nuclear and magnetic terms, can satisfy the condition of being unitarily equivalent to its transpose, thereby guaranteeing reciprocity under specific orientations of the magnetic field.

## 1. Introduction

In recent years, the mathematical framework of complex symmetric operators (CSOs) *has* emerged as a cornerstone in the description of non-Hermitian physical phenomena. Recent studies have demonstrated their pivotal role in identifying semiclassical resonances [1], managing gain-loss balances in PT-symmetric quantum systems [2], and uncovering novel topological phases in non-Hermitian photonics [3,4]. Crucially, the transition from Hermitian to complex symmetric representations reflects the fundamental shift from reversible dynamics to irreversible processes, providing a natural language for systems where time-reversal symmetry is modified or broken. This symmetry is deeply intertwined with the Onsager reciprocity relations and the microscopic foundations of entropy production [5]. In this context, Deák and Fülöp [6] provided a comprehensive unified treatment of reciprocity across quantum, electromagnetic, and other wave scattering phenomena, establishing that reciprocity is a fundamental consequence of the underlying symmetry properties of the system’s scattering operators. By examining the interplay between complex symmetry and the spectral properties of non-equilibrium systems, one can gain deeper insights into the thermodynamic arrow of time and the bounds of entropy generation in dissipative environments [7]. Building upon these developments, the present work aims to further elucidate the role of complex symmetry in quantum mechanical scattering problems, establishing a robust link between operator theory and the principles of statistical irreversibility.

Symmetry is a fundamental issue in physics that can be interpreted as a concept of balance or patterned self-similarity [8]. The physical system itself, as well as a theoretical model, may have symmetry properties [6]. According to the Oxford online dictionary, symmetry is a law or operation where a physical property or process has an equivalence in two or more directions, or events/actions are balanced/equal in some way. A synonym to the term symmetry is invariance, expressing the fact that a special operation (called symmetry transformation)—like a geometric transformation, a change of polarization, parity, charge, the arrow of time, etc.—does not change some particular property of the system. Depending on the studied system, symmetry may have various measures and operational definitions. Symmetry transformations were defined by Wigner [9] as general operators preserving the modulus of scalar products of the vectors of the Hilbert space representing the physical states. Further, the symmetry theorem of Wigner states that any symmetry transformation can be represented by either a unitary linear or an antiunitary (isometric conjugate-linear) operator.

In practice, antiunitary cases are much less frequently encountered than unitary ones [6]. Two well-known antiunitary symmetries are charge conjugation and time reversal. We discuss validity of antiunitary symmetries in a general scattering problem by giving a condition for the scattering potential. It results that the complex symmetric property of the scattering potential indicates the existence of an antiunitary symmetric property of the complex transition amplitude.

While the present work specifically focuses on the operational conditions for reciprocity via antiunitary symmetries, it is situated within a rapidly expanding broader field. Recently, related generalized non-Hermitian symmetries—such as pseudo-Hermiticity and generalized PT symmetry—have been increasingly applied to diverse phenomena, including quantum state discrimination [10] and complex entropy dynamics [11].

Finally, we apply the developed formalism to a concrete physical system: polarized neutron reflectometry (PNR). Neutron scattering offers an ideal platform for testing these symmetry principles due to the interplay between the isotropic nuclear potential and the anisotropic magnetic interaction. It is important to emphasize that in this system, time-reversal symmetry is violated by two distinct mechanisms: explicitly by the external magnetic field, and implicitly by the complex nuclear optical potential, which accounts for absorption (dissipation). We show that despite this double violation, a generalized antiunitary symmetry—reciprocity—remains intact. In the context of PNR, we explicitly construct the unitary operators required to establish the antiunitary symmetry of the scattering amplitude.

## 2. Materials and Methods: Antiunitary Operators and Conjugations

In this section we revisit antiunitary operators, briefly summarizing some definitions. In a separable complex Hilbert space H, an operator U:H⟶H is called unitary if it is isometric,

(1) $$\left\|U\psi \right\|=\left\|\psi \right\|,\;\;\psi \in H$$

and linear:

(2) $$U\left(\lambda_{1}\psi_{1}+\lambda_{2}\psi_{2}\right)=\lambda_{1}U\psi_{1}+\lambda_{2}U\psi_{2},\;\lambda_{1},\lambda_{2}\in C,\;\psi_{1},\psi_{2}\in H$$

For antiunitary operators K:H⟶H, isometry remains valid:

(3) $$\left\|K\psi \right\|=\left\|\psi \right\|,\;\psi \in H$$

while linearity is replaced by antilinearity:

(4) $$K\left(\lambda_{1}\psi_{1}+\lambda_{2}\psi_{2}\right)=\lambda_{1}^{*}K\psi_{1}+\lambda_{2}^{*}K\psi_{2},\;\lambda_{1},\lambda_{2}\in C,\;\psi_{1},\psi_{2}\in H$$

where * denotes usual complex conjugation. In both cases, isometry implies the existence of the inverse operator, and the inverse proves to coincide with the adjoint,

(5) $$U^{-1}=U^{†},\;K^{-1}=K^{†}$$

which are again unitary and antiunitary, respectively. With the aid of the so-called polarization identity, from the isometric property one finds for any $\psi_{1},\psi_{2}\in H$

(6) $$\left(U\psi_{1},U\psi_{2}\right)=\left(\psi_{1},\psi_{2}\right)$$

and

(7) $$\left(K\psi_{1},K\psi_{2}\right)=\left(\psi_{2},\psi_{1}\right)$$

respectively. From the property expressed in Equation (7)—namely, that antiunitary operators reverse the order of the arguments in the scalar product—it follows that they likewise interchange the initial and final states in scattering processes, thereby endowing antiunitary symmetry transformations with a particular conceptual significance.

The most frequently treated antiunitary operators are the involutive ones, and are called conjugations. Namely, an antiunitary operator *C* is a conjugation if it is involutive,

(8) $$C^{2}=I$$

with I denoting the identity operator of the Hilbert space H. Choosing an arbitrary antiunitary operator—for later purposes, let it actually be a conjugation C—any antiunitary K can be written in the form

(9) K=UC,

where U is unitary. The most well-known example for a conjugation operator is the standard complex conjugation

(10) $$J\psi =\psi^{*}$$

of complex functions ψ in a $L^{2}$ Hilbert space.

It is beneficial to recall that, for n×n complex matrices—the operators of the Hilbert space $C^{n}$—the adjoint is the transpose of the complex conjugate:

(11) $$M^{†}={\left(M^{*}\right)}^{T}$$

With the aid of the standard complex conjugation operator J we can also express $M^{*}$ as

(12) $$M^{*}=JMJ^{-1}$$

(11) can be reformulated as

(13) $$M^{†}={\left(JMJ^{-1}\right)}^{T}$$

yielding

(14) $$M^{T}={\left(JMJ^{-1}\right)}^{†}$$

This enables us to define the transpose of an operator V of an arbitrary Hilbert space, with respect to a fixed conjugation operator C, as $V^{T}≔{\left(CVC^{-1}\right)}^{†}$. To this end, we recall that a complex matrix M is a self-transpose one (also called complex symmetric) if

(15) $$M^{T}=M,$$

and this definition can be used now for the operator V of the Hilbert space

(16) $$V^{T}={\left(CVC^{-1}\right)}^{†}$$

## 3. Antiunitary Symmetry in the General Scattering Problem

A large class of physical processes can be described in the framework of scattering theory [12,13], which is a theoretical sub-model inside quantum mechanics, but can be interpreted in classical electrodynamics and in the classical mechanics of elastic waves as well. Concerning scattering theory, we use notations, conventions, and standard results from [12,13].

Let $H_{0}$ be a self-adjoint Hamiltonian, which, for simplicity, will be called a free Hamiltonian although it need not really describe a free quantum/wave propagation—for example, neutrons emitted by a source may travel through a guiding magnetic field. Furthermore, let a potential V describe a scatterer. V is not assumed to be self-adjoint, which allows absorption effects to be incorporated.

For any two eigenstates $u_{\alpha },$ $u_{\beta }$ of $H_{0}$ with eigenvalue $E_{\alpha }=E_{\beta }$, the $u_{\alpha }\to u_{\beta }$ complex elastic scattering transition amplitude reads and satisfies

(17) $$\left\langle \beta |T|\alpha \right\rangle ≔\left(u_{\beta },\;V\chi_{\alpha }^{+}\right)=\left(\chi_{\beta }^{T-},\;{Vu}_{\alpha }\right)$$

with $\chi_{\alpha }^{+}$ and $\chi_{\beta }^{T-}$ being such $E_{\alpha }=E_{\beta }$ eigenvalued eigenstates of $H=H_{0}+V$ and its adjoint $H^{†}$ respectively that $u_{\alpha }$ and $u_{\beta }$ are their asymptotically incoming (‘+’ sign) or outgoing (‘−’ sign) part [12,13].

In a scattering situation as described before, let us assume that an antiunitary operator K commutes with the free Hamiltonian

(18) $$KH_{0}K^{-1}=H_{0}$$

and also that it connects the potential *V* with its adjoint as

(19) $$KVK^{-1}=V^{†}$$

Then, for the full Hamiltonian $H=H_{0}+V,$ we have

(20) $$KHK^{-1}=H^{†}.$$

We note that the scattering potential *V* and, consequently, the full Hamiltonian *H* are not assumed to be Hermitian.

Considering the $u_{\alpha }\to u_{\beta }$ elastic scattering transition amplitude, let us introduce the notations

(21) $$u_{\bar{\alpha }}≔{Ku}_{\alpha },\;u_{\bar{\beta }}≔{Ku}_{\beta }$$

K commutes with $H_{0}$ and we are treating an elastic process so

(22) $$E_{\alpha }=E_{\beta }=E_{\bar{\alpha }}=E_{\bar{\beta }}≔E$$

Applying (19) and (17), one finds

(23) $$K\chi_{\alpha }^{\pm }=\chi_{\bar{\alpha }}^{T\mp },\;K\chi_{\beta }^{\pm }=\chi_{\bar{\beta }}^{T\mp }$$

In other words, *K* maps a scattering process to a ‘‘reversed’’ one. Hence, for the transition amplitude, we obtain

(24) $$\left\langle \beta |T|\alpha \right\rangle =\left(u_{\beta },V\chi_{\alpha }^{+}\right)=\left(KV\chi_{\alpha }^{+},Ku_{\beta }\right)=\left(KVK^{-1}K\chi_{\alpha }^{+},Ku_{\beta }\right)=\;=\left(V^{†}K\chi_{\alpha }^{+},Ku_{\beta }\right)=\left(K\chi_{\alpha }^{+},VKu_{\beta }\right)=\left(\chi_{\bar{\alpha }}^{T-},Vu_{\bar{\beta }}\right)=\left\langle \bar{\alpha }|T|\bar{\beta }\right\rangle$$

where (7), (17), (19), (21), (23), and again (17) have been utilized, in turn [6].

This result (24) is the reciprocity theorem for the transition amplitude [6], which relates a scattering process to a “reversed one”, by exchanging the transformed initial and final states, $u_{\alpha }\to u_{\beta }$ to $u_{\bar{\beta }}\to u_{\bar{\alpha }}$. The left–right interchanging property (7) of the antiunitary operator makes this “reciprocal” relation possible, explaining also why an antiunitary transformation differs from the usual unitary ones. It is clear that (24) is only valid if (19) is fulfilled. Symmetry means again that the transition amplitude (17) is invariant for the antiunitary transformation K.

The main question remains; how can we check whether requirement (19) is met for a given scattering potential? In other words, is there an appropriate antiunitary operator K for a given potential V that satisfies condition (19)? Typically, it is easier to check K against the free Hamiltonian and the less easy task is to investigate the validity of (19). To get closer to the answer to the latter question, let us choose an auxiliary conjugation C arbitrarily, with the aid of which we describe the various possible Ks by the various possible Us via (9). This way, we can rewrite (19) as

(25) $$V={\left(KVK^{-1}\right)}^{†}={\left(UCVC^{-1}U^{-1}\right)}^{†}={U\left(CVC^{-1}\right)}^{†}U^{-1}$$

and, finally, according to (16), the conditiofn (19) is rewritten as

(26) $$V=UV^{T}U^{-1}$$

The difficult-to-handle condition (19) is thus transformed to expression (26) so that linear operators are included only. According to expression (26), our symmetry condition can be expressed as follows: the scattering potential should be unitary equivalent to its transposed (UET) one.

### Application of the Theory: Polarized Neutron Reflectometry

To illustrate the interplay between non-Hermitian dynamics and reciprocity, we apply our formalism to PNR. In this case, the presence of a magnetic field breaks the conventional time-reversal symmetry, yet we demonstrate that an antiunitary symmetry is preserved, ensuring the validity of the reciprocity relations.

The general consequence of the existence of an antiunitary symmetry, as given by Equation (9), is the reciprocity relation expressed in Equation (24). We can verify the validity of this symmetry by examining the scattering potential *V*. In PNR, the scattering potential *V* is composed of a complex isotropic nuclear term and an anisotropic magnetic term [14]. Specifically, we must determine whether the potential *V* is unitarily equivalent to its transpose (UET) according to the condition in Equation (26).

In the quantum mechanical description of neutrons, a two-component wave function is employed:

(27) $$\psi =\left(\begin{array}{c} \psi_{1}\\ \psi_{2} \end{array}\right),$$

where $\psi_{1}$ and $\psi_{2}$ are spatially square-integrable complex functions. The antiunitary symmetry operators, following Equations (9) and (10), take the form *K* = *UJ*. In cases of specular reflection or transmission, the spatial propagation is fixed by the boundary conditions; thus, our analysis focuses on the polarization space.

According to [14], the scattering potential is given by

(28) $$V=4\pi N\sum_{i}\alpha_{i}b_{i}\sigma_{0}-\frac{2m}{\hbar^{2}}g\mu_{N}\vec{\sigma }\cdot \vec{B},$$

Here, *m* is the neutron mass, the index *i* denotes the different types of scattering centers, $\alpha_{i}$ is the relative abundance, and *b_i_* is the complex nuclear scattering length of the *i*th nucleus. *N* represents the number density of scattering centers. Furthermore, *g* = 1.9132, $\mu_{N}=5.050\times 10^{-27}$ Am^2^ is the neutron magnetic moment, $\vec{\sigma }=\left(\sigma_{1},\sigma_{2},\sigma_{3}\right)$ is the vector of Pauli matrices, and $\vec{B}=\left(B_{1},B_{2},B_{3}\right)$ is the homogeneous mean magnetic induction within a layer, defined in a coordinate system spanned by unit vectors **e**_1_, **e**_2_, **e**_3_.

Using the standard Pauli matrices

(29) $$\sigma_{0}=\left(\begin{array}{cc} 1 & 0\\ 0 & 1 \end{array}\right),\;\sigma_{1}=\left(\begin{array}{cc} 0 & 1\\ 1 & 0 \end{array}\right),\;\sigma_{2}=\left(\begin{array}{cc} 0 & -i\\ i & 0 \end{array}\right),\;\sigma_{3}=\left(\begin{array}{cc} 1 & 0\\ 0 & -1 \end{array}\right),$$

the potential (28) can be written in matrix form as

(30) $$V=4\pi N\sum_{i}\alpha_{i}b_{i}\left(\begin{array}{cc} 1 & 0\\ 0 & 1 \end{array}\right)-\frac{2m}{\hbar^{2}}g\mu_{N}\left(\begin{array}{cc} B_{3} & B_{1}-iB_{2}\\ B_{1}+iB_{2} & -B_{3} \end{array}\right).$$

The first term, the nuclear potential term, is typically complex, incorporating absorption effects. This non-Hermiticity constitutes a fundamental violation of time-reversal symmetry, distinct from the symmetry breaking induced by the magnetic field. Consequently, standard time-reversal arguments fail, necessitating the use of the broader antiunitary symmetry framework to establish reciprocity. The scattering potential (30) is not complex symmetric (self-transpose) due to the anti-symmetric property of *σ*_2_. To establish antiunitary symmetry, we must verify Equation (26): does a unitary operator *U* exist such that $V=UV^{T}U^{-1}$?

Transposing the matrices in Equation (30) effectively changes the sign of the imaginary part in the off-diagonal elements, which corresponds to reversing the sign of the second component of the magnetic field, $B_{2}=\vec{B}\cdot e_{2}$. Geometrically, this transposition is equivalent to a reflection across the plane spanned by **e**_1_ and **e**_3_; it preserves the 1st and 3rd components of the magnetic field vector while reversing the 2nd.

Since the magnetic field exists in $R^{3}$, we can find a rotation that transforms the vector $\left(B_{1},{-B}_{2},B_{3}\right)$—representing the transposed potential $V^{T}$—back to the original potential vector $\left(B_{1},B_{2},B_{3}\right)$. Consider a plane containing the vector $\left(B_{1},{-B}_{2},B_{3}\right)$. This plane intersects the **e**_1_–**e**_3_ plane along a line. Let the unit vector **n** of this line be the axis of rotation, and let the rotation angle *φ* be twice the angle between the two planes. The required unitary transformation is given by:

(31) $$U=\;e^{i\delta }\left(\sigma_{0}\cos\frac{\varphi }{2}-in\cdot \vec{\sigma }\sin\frac{\varphi }{2}\right),$$

where 0≤δ≤2π is an arbitrary phase [10].

Having identified the transformation, we note that experimentally verifying the interchange of initial and final states (changing source and detector positions) is often impractical at large facilities. Instead, it is more feasible to rotate the sample. For elastic scattering such as PNR, this corresponds to a 180° rotation of the sample around the momentum transfer vector. We denote this unitary rotation operator by $U_{\pi }$.

In reflectometry, the scattered field $\psi_{S}$ is related to the incident field $\psi_{0}$ by

(32) $$\psi_{S}=S\psi_{0},$$

where *S* is the 2 × 2 complex scattering matrix (common notation for reflectivity *R* or transmissivity *T* matrices) acting on the polarization space. In the polarization basis defined by

(33) $$e_{+}=\left(\begin{array}{c} 1\\ 0 \end{array}\right),\;e_{-}=\left(\begin{array}{c} 0\\ 1 \end{array}\right).$$

the matrix elements of *S* satisfy

(34) $$S_{\mu \nu }=\left(e_{\mu },Se_{\nu }\right)=\left(e_{\nu },S^{T}e_{\mu }\right)=\left(e_{\nu },U^{-1}SUe_{\mu }\right)=\left(Ue_{\nu },SUe_{\mu }\right)$$

where we utilized the unitary property $U^{-1}=U^{†}$ and the assumed antiunitary symmetry of *S*. The indices μ, ν=+,− denote the components of polarization vectors.

To provide a practical formula for experimental verification, we introduce the rotation operator $U_{\pi }$. The relation becomes

(35) $$S_{\mu \nu }=\left(Ue_{\nu },SUe_{\mu }\right)=\left(U_{\pi }Ue_{\nu },S_{\pi }U_{\pi }Ue_{\mu }\right),\;$$

where $S_{\pi }=U_{\pi }SU_{\pi }^{-1}$ is the scattering matrix of the rotated sample. This result implies that under antiunitary symmetry, the scattering matrix elements are related to those of the rotated system, with polarization states transformed by the composite operator $U_{\pi }U$.

#### Specific Example

Consider a multilayer scatterer where each layer has parallel surfaces and a homogeneous in-plane magnetic field. Geometrically, the layer surfaces are parallel to the **e**_1_—**e**_2_ plane. If we define the transformation such that the rotation axis is parallel to **e**_1_, the unitary operator *U* corresponds to a 180° rotation around **e**_1_. Following Equation (31), we can choose $U=\sigma_{1}$ (with appropriate phase). The sample rotation $U_{\pi }$ is typically a 180° rotation around the surface normal (parallel to **e**_3_), so $U_{\pi }=\sigma_{3}$.

Substituting these into the composite transformation,

$$U_{\pi }U=\sigma_{3}\sigma_{1}=i\sigma_{2}.$$

Applying this to the basis vectors in Equation (33), we find $\sigma_{2}e_{+}=ie_{-}$ and $\sigma_{2}e_{-}=-ie_{+}$. Consequently, the scattering matrix elements relate as follows:

(36) $$S_{++}={\left(S_{\pi }\right)}_{--},\;\;S_{+-}={-\left(S_{\pi }\right)}_{-+}\;S_{--}={\left(S_{\pi }\right)}_{++},\;\;S_{-+}={-\left(S_{\pi }\right)}_{+-}$$

Equation (36) is the consequence of antiunitary symmetry. It is important to note that unitary symmetry like rotation is unable to exchange an incoming polarization with an outgoing one. The general antiunitary operator was expressed in Equation (9) as product of a unitary operator and an antilinear involution, being in our special case a rotation and the standard complex conjugation, respectively.

## 4. Discussion of Further Aspects of Antiunitary Symmetry Property of the Transition Amplitude

### 4.1. Experimental Evidence

The aforementioned theoretical results have already been experimentally verified using nuclear resonance scattering of synchrotron radiation. By adjusting the experimental geometry, both reciprocity and a non-reciprocity spanning three orders of magnitude were observed in the measured intensities, in full agreement with theoretical predictions [15]. Notably, the presence of the magneto-optic Faraday effect does not automatically result in non-reciprocity. Given that non-reciprocal devices constitute a critical class of optical components, further applications in the field of optics are anticipated.

For polarization-dependent scattering, reciprocity can also be violated in the phase of the scattering amplitude, a phenomenon significantly more challenging to observe experimentally than violations in magnitude. Leveraging the advantageous properties of nuclear resonance scattering of synchrotron radiation, a maximal phase-reciprocity violation of 180° was successfully measured [16]. The experimental configuration was designed based on a generalized reciprocity theorem derived through the use of CSOs, paving the way for the development of novel types of reciprocity-based devices.

We have demonstrated that in PNR studies of thin films, provided that all magnetic field vectors are coplanar, an antiunitary symmetry for the potential can always be found. Indeed, in such cases, there exists a rotation that maps the common plane of the magnetic fields to the **e**_1_–**e**_3_ plane; consequently, due to the absence of the *y*-components of the magnetic fields, the potential becomes complex symmetric. The converse is also true: if the magnetic fields within the layers are non-coplanar, the investigated antiunitary symmetry does not manifest, and relations analogous to Equation (36) are not obtained. In practice, there exist thin-film structures where the internal magnetic configurations can be reoriented via an external magnetic field. In such instances, for example, the magnetic field in certain layers may cant out of the film plane, causing the structure to transition from a reciprocal to a non-reciprocal system. The investigation of such exotic non-reciprocal devices is currently a field of intense research. A deeper theoretical understanding of antiunitary transformations and symmetries may provide significant impetus for the development of novel non-reciprocal neutron optical devices.

### 4.2. Mathematical Implications

Why the form (26) is worth considering becomes apparent when we turn towards the mathematical literature. Indeed, there is increasing interest in conjugation operators and, more generally, in antiunitary ones [17,18,19,20,21,22,23,24]. Here, let us only mention some simple special cases. To this end, we recall that a complex matrix *M* is a self-transpose one (complex symmetric) if (15) is fulfilled. It is evident that self-transpose matrices are UET. What results is that any matrix M that is unitarily equivalent to a complex symmetric matrix (UECSM),

(37) $${\left(\tilde{U}M{\tilde{U}}^{-1}\right)}^{T}=\tilde{U}M{\tilde{U}}^{-1}\;\;(\mathrm{for}\;\mathrm{some}\;\mathrm{unitary}\;\tilde{U})$$

also proves to satisfy $M=UM^{T}U^{-1}$, with $U={\left({\tilde{U}}^{T}\tilde{U}\right)}^{-1}$. Further, in his problem book, P.R. Halmos asks whether every square complex matrix is UET [24]. In the discussion, Halmos introduces the single counterexample

(38) $$M=\left(\begin{array}{ccc} 0 & 1 & 0\\ 0 & 0 & 2\\ 0 & 0 & 0 \end{array}\right)$$

and proves that it is not UET. The question of whether a given operator is actually a complex symmetric operator is more subtle than it first appears. For instance, one can show that among the matrices

(39) $$\left(\begin{array}{ccc} 0 & 7 & 0\\ 0 & 1 & 2\\ 0 & 0 & 6 \end{array}\right),\left(\begin{array}{ccc} 0 & 7 & 0\\ 0 & 1 & 3\\ 0 & 0 & 6 \end{array}\right),\left(\begin{array}{ccc} 0 & 7 & 0\\ 0 & 1 & 4\\ 0 & 0 & 6 \end{array}\right),\left(\begin{array}{ccc} 0 & 7 & 0\\ 0 & 1 & 5\\ 0 & 0 & 6 \end{array}\right),\left(\begin{array}{ccc} 0 & 7 & 0\\ 0 & 1 & 6\\ 0 & 0 & 6 \end{array}\right)$$

only the fourth matrix is UECSM [17,19].

Especially relevant to our situations is the paper by Garcia and Tener, where the authors study exactly condition (26), in Hilbert spaces $C^{n}$, i.e., for n×n complex matrices V, U. They present a necessary and sufficient condition for those Vs that fulfill (26) with some appropriate unitary U—in other words, which Vs are UET. This necessary and sufficient condition [19] of the finite dimensional case can be directly applicable for several infinite dimensional Hilbert space situation when, for practical purposes and applications, one restricts the search for U (search for K) to some special product forms. In addition, the known finite dimensional result paves the way for the corresponding infinite dimensional theorem to come.

As an example, we close this section on the mathematical literature by quoting a finding by Garcia and Tener in Ref. [19]: For n≤7, any n×n complex matrix that is UET is also UECSM, meaning it can be brought into self-transpose form by a certain unitary transformation. Surprisingly, the naive assertion that a matrix is UET if and only if it is unitarily equivalent to a complex symmetric matrix holds for matrices 7×7 and smaller, but fails for matrices 8×8 and larger [19].

## 5. Conclusions

The antiunitary symmetry operations are not very often used in physics, although interesting relationships follow when they exist. In scattering experiments the existence of an antiunitary operator *K* that commutes with the free Hamiltonian and transforms the scattering potential to its transpose indicates the equivalence of any scattering amplitude $\left\langle \beta |T|\alpha \right\rangle$ to another scattering amplitude, namely, to $\left\langle K\alpha |T|K\beta \right\rangle$. This left–right interchange of incoming and outgoing states, given by reciprocity, in important experimental applications, means that a scattering amplitude is related to another one where the source and the detector are interchanged. Remarkably, reciprocity can hold for non-self-adjoint systems (systems with absorption), too.

Our results demonstrate that reciprocity is a robust property that can survive even when time-reversal symmetry is broken by both magnetic interactions and dissipative (absorptive) nuclear processes.

To find antiunitary symmetry (reciprocity) operators for a given system is a delicate problem in general. The relationship established here to a recently developing area of mathematics is expected to give an impetus to finding reciprocity operators to more physical systems, resulting in valuable applications.

Our analysis of the polarized neutron reflectometry case confirmed that even in the presence of magnetic fields—where time-reversal symmetry is traditionally broken—an antiunitary symmetry can be recovered. By identifying the specific unitary transformation that maps the potential to its transpose, we demonstrated how reciprocity is manifested.

## Data Availability

Data are contained within the article.

## Abbreviations

The following abbreviations are used in this manuscript:

CSO	Complex Symmetric Operator
UET	Unitary Equivalent to its Transposed
UECSM	Unitarily Equivalent to a Complex Symmetric Matrix
PNR	polarized neutron reflectometry
